# Dual-Plasma Discharge Tube for Synergistic Glioblastoma Treatment

**DOI:** 10.3390/cancers17122036

**Published:** 2025-06-18

**Authors:** William Murphy, Alex Horkowitz, Vikas Soni, Camil Walkiewicz-Yvon, Michael Keidar

**Affiliations:** Micropropulsion and Nanotechnology Laboratory (MPNL), School of Engineering and Applied Science, George Washington University, 800 22nd St. NW, Suite 3100, Washington, DC 20052, USA; murphyw@gwu.edu (W.M.); ahorkowitz@gwu.edu (A.H.); camilyvon@gwu.edu (C.W.-Y.)

**Keywords:** plasma, cold plasma, cancer, discharge tube, oncology, glioblastoma, cold atmospheric plasma, cancer therapy, nonthermal plasma, Electromagnetic field (non-invasive) therapy

## Abstract

Background: Glioblastoma (GBM) resists current therapies due to its rapid proliferation, diffuse invasion, and heterogeneous cell populations. We previously showed that a single cold atmospheric plasma discharge tube (DT) reduces GBM viability via broad-spectrum electromagnetic (EM) emissions. Here, we tested whether two DTs arranged in a helmet configuration could generate overlapping EM fields to amplify the anti-tumor effects without thermal injury. Methods: The physical outputs of the single- and dual-DT setups were characterized by infrared thermography, broadband EM field probes, and oscilloscope analysis. Human U87-MG cells were exposed under the single or dual configurations. The viability was quantified with WST-8 assays mapped across 96-well plates; the intracellular reactive oxygen species (ROS), membrane integrity, apoptosis, and mitochondrial potential were assessed by multiparametric flow cytometry. Our additivity models compared the predicted versus observed dual-DT cytotoxicity. Results: The dual-DT operation produced constructive EM interference, elevating electric and magnetic field amplitudes over a broader area than either tube alone, while temperatures remained <39 °C. The single-DT exposure lowered the cell viability by ~40%; the dual-DT treatment reduced the viability by ~60%, exceeding the additive predictions. The regions of greatest cytotoxicity co-localized with the zones of highest EM field overlap. The dual-DT exposure doubled the intracellular ROS compared with single-DT and Annexin V positivity, confirming oxidative stress-driven cell death. The out-of-phase operation of the discharge tubes enabled the localized control of the treatment regions, which can guide future treatment planning. Conclusions: Two synchronously operated plasma discharge tubes synergistically enhanced GBM cell killing through non-thermal mechanisms that coupled intensified overlapping EM fields with elevated oxidative stress. This positions modular multi-DT arrays as a potential non-invasive adjunct or alternative to existing electric-field-based therapies for glioblastoma.

## Simple Summary

Glioblastoma is an aggressive, high grade brain tumor that is difficult to treat effectively. In our study, we investigated a novel non-invasive treatment for glioblastoma using two cold atmospheric plasma discharge tubes arranged in a helmet-like configuration. We found that two plasma tubes could create overlapping electromagnetic fields that increased tumor cell death compared to using just one tube. We correlated cancer cell death and cellular oxidative stress mechanisms with the electromagnetic field strength emitted from the device, but they were not correlated with heat generation (negligible thermal effects). Our findings highlight the potential to use this dual-plasma approach as a non-invasive, energy-directed treatment for glioblastoma.

## 1. Introduction

Glioblastoma (GBM) is a grade IV astrocytoma and one of the most aggressive primary brain tumors, defined by a diffuse infiltration into the surrounding brain tissue and a notorious resistance to the conventional therapies. Even with maximal surgery, high-dose chemotherapy, and radiation, the prognosis remains poor; the median survival is on the order of only 15 months, and the five-year survival rates linger below 5% [1,2]. The infiltrative growth of GBM makes complete surgical resection virtually impossible, and residual invasive cells rapidly drive recurrence. GBM cells exhibit intrinsic and acquired resistance mechanisms (e.g., DNA repair, anti-apoptotic signaling) that blunt the effectiveness of cytotoxic chemotherapy and radiation, underscoring the urgent need for new treatment modalities that can overcome these challenges.

Recent advances have explored non-invasive biophysical therapies such as Tumor Treating Fields (TTF). TTF employ alternating electric fields in the kilohertz range delivered through transducer arrays on the scalp to disrupt mitotic spindle assembly, thereby inhibiting tumor cell division and inducing apoptosis [3]. In the pivotal EF-14 trial, the addition of TTF to standard temozolomide therapy significantly extended the median overall survival from 16.0 to 20.9 months and the progression free survival from 4.0 to 6.7 months. Systemic adverse events were no different between the groups, while mild to moderate skin toxicity was observed in 52% of the patients in the TTF group vs. 0% in the control arm [4]. However, TTF operate in a relatively narrow frequency window (~200 kHz) optimized for mitosis interference and use a uniform field geometry. These constraints may limit their impact on the heterogeneous cell populations in GBM, particularly the cells outside of active division or in microenvironment niches that confer additional therapy resistance. In essence, while TTF have proven beneficial, their mechanism (focused primarily on mitotic disruption) may not fully address the full biology of GBM, prompting interest in more comprehensive approaches. TTF application also requires long treatment times (18–24 h), creating unwanted side effects [3,4]. On the other hand, a direct comparison of plasma treatment and TTF showed that the same effect can be achieved with a much shorter treatment time and much lower energy [5].

Cold atmospheric plasma (CAP) has emerged as a potential cancer therapy that could complement or be an alternative to traditional treatments. CAP is an ionized gas near room temperature generated by applying high voltage to a gas (e.g., helium or argon) at atmospheric pressure, producing a cocktail of reactive species along with electromagnetic (EM) emissions. Unlike the static, single-frequency fields of TTF, CAP delivers a broad spectrum of factors: reactive oxygen and nitrogen species (RONS), charged particles, ultraviolet and visible light photons, and time-varying EM fields spanning frequencies from kilohertz to gigahertz [6,7]. These diverse outputs can simultaneously target multiple cellular structures and pathways. CAP has been shown in experimental models to induce oxidative stress, disrupt cell membranes and organelles, depolarize mitochondrial membranes, and activate programmed cell death in cancer cells [6,7]. Such multi-modal effects give CAP the ability to attack tumor cells on several fronts (inducing DNA damage, lipid peroxidation, and protein oxidation), which is particularly important for aggressive cancers such as GBM that exploit numerous survival pathways. CAP’s mechanisms are not limited to cell division; even quiescent or therapy-resistant GBM cells (such as stem-like or hypoxic cells) may be vulnerable to plasma-induced stress [7].

An extensive amount of preclinical evidence supports CAP’s potent anti-glioblastoma activity. CAP exposure can generate a surge of intracellular RONS within tumor cells, overwhelming their antioxidant defenses and causing oxidative DNA damage and lipid peroxidation, which trigger apoptotic signaling [8,9]. Reactive oxygen species (ROS), similar to hydrogen peroxide, induce oxidative stress, while reactive nitrogen species (RNS), such as nitric oxide, modulate signaling and proteins, causing functional disruptions. For instance, the direct CAP treatment of GBM cells has been shown to cause DNA double-strand breaks and deactivate key pro-survival kinases such as AKT, leading to apoptosis and loss of clonogenicity in vitro [9]. At the same time, CAP perturbs mitochondrial function; experiments have observed a collapse of the mitochondrial membrane potential in plasma-treated glioma cells, consistent with the initiation of the intrinsic (mitochondrial) apoptotic pathway [8]. Several studies have also noted increases in caspase activation, PARP cleavage, and other hallmarks of apoptosis in GBM and other cancer cell lines following CAP treatment, confirming that plasma-induced damage reaches the level of irreversible cell death commitments [9]. CAP’s cytotoxicity appears to exhibit a degree of selectivity for cancer cells. Normal astroglial cells and other non-malignant controls show minimal damage under equivalent CAP exposure conditions, whereas GBM cells undergo significant apoptosis and growth inhibition [10,11]. This selectivity has been attributed to cancer cells’ higher baseline ROS levels and compromised redox homeostasis, making them more susceptible to further oxidative stress, as well as differences in membrane composition and repair capacity [10,11]. Together, these effects position CAP as a uniquely comprehensive modality for attacking GBM cells on multiple biological fronts.

It is notable that the anti-tumor effects of CAP arise from both chemical and physical effectors. On the chemical side, plasma generates a variety of long-lived RONS that can diffuse into liquids to create “plasma-activated media” (PAM) [12]. Tanaka et al. demonstrated that PAM could selectively kill glioblastoma cells in vitro, in part by downregulating the AKT survival signaling pathway and promoting apoptosis [13]. This finding indicates that CAP’s reactive chemical species alone are capable of eliciting anti-cancer effects. On the other hand, CAP also delivers physical stimuli, such as electromagnetic fields, thermal and acoustic perturbations, and UV and visible light radiation, that can act on cells even without direct plasma-to-cell contact. Contained CAP devices such as the discharge tube (DT) trap the plasma chemistry inside a dielectric vessel, whereby only the physical factors are able to affect the external target (cell culture sample or in vivo model). Foundational research using the plasma discharge tube (DT) design demonstrated the ability to induce cell death in GBM cells across an insulating barrier [14,15,16,17]. These results indicate that physically emitted factors penetrated the barrier and activated cell death pathways in the absence of plasma generated RONS [14,15,16,17]. TTF, PDT, and other plasma devices have also shown a particular ability to sensitize GBM to temozolomide treatment [18,19,20,21]. Most interesting is the in vivo data published by Yao et al. in 2022 demonstrating the in vivo effectiveness of PDT to elicit synergistic effects when combined with temozolomide [21]. This effect underscores the unique contribution of CAP’s physical components, which can stress cells through physical mechanisms, mainly electromagnetic emissions, distinct from the chemical RONS-mediated pathways. By influencing charged biomolecules, membrane potentials, and possibly intracellular calcium dynamics or other field-sensitive processes, these physical factors add another dimension to CAP therapy. Recognizing this dual physical and chemical nature of CAP is important for designing treatment strategies that effectively treat cancers [13,14]. Indeed, studies from our group and others continue to explore how these pathways can be modulated to maximize cancer cell killing while minimizing off-target effects.

While the effects of ionizing radiation (UV, X-ray, gamma ray) on biological systems are well known and have been used in biomedical applications for decades, the effects of non-ionizing radiation (hertz to optical frequencies) on biology have been demonstrated but are less understood. We provide an overview of the most pertinent effects on biological systems here, and readers should refer to the 2022 review article by Romeo et al. for a deeper understanding of EM effects and the mechanisms involved [22].

Non-ionizing EM radiation in the RF and visible light frequencies has been shown to cause oscillations in the plasma membrane [23] and the activation of embedded protein channels such as TRPC1 via pulsed electromagnetic field exposure [24]. The direct or indirect stimulation of membrane bound receptors and ion channels typically results in an alteration of the intracellular calcium ion concentrations. Large influxes of calcium ions are known to affect mitochondrial function in the formation of mitochondrial permeability transition pores, resulting in the further release of mitochondrial ROS and a reduction in ATP production [25,26]. With the proximity of mitochondria to cytoskeletal elements such as microtubules, microtubule assemblies are also highly sensitive to intracellular ROS and calcium ion concentrations [27]. While the effects of ROS and calcium ions on the cytoskeleton are an indirect effect of EM stimulation, the direct effects of the oscillation of biopolymers including f-actin and tubulin, making up actin and microtubule fibers, respectively, can also be induced by RF EM waves [28]. Additionally, native ionic currents and oscillations on microtubules have been measured in the kHz frequency regime and may be resonantly affected by EM sources of a similar frequency, such as plasma devices with a kHz discharge frequency [29,30,31].

Given CAP’s anti-tumor effects, a next possible development is to amplify its delivery to the tumor. One approach is the use of multiple plasma discharge tubes simultaneously to increase the treatment volume and to achieve overlapping fields for a potential synergistic impact. A single discharge tube (DT) device can affect cells in close proximity (1–4 cm) via emitted EM fields and other physical effectors, but its reach is limited in space and intensity [16]. We hypothesize that operating two CAP sources in tandem, arranged around the target similar to a helmet, will create a more complex electromagnetic field topology that enhances the overall cytotoxic stimulus. The superposition of the EM fields from dual DTs could produce regions of higher field amplitude through constructive interference and expose cells to a wider range of frequencies and field orientations, possibly stressing cellular structures beyond what one device can accomplish alone. The use of multiple plasma discharge tubes is proposed as a means to achieve spatially enhanced and synergistic bioelectric and biochemical effects on GBM.

In this work, we introduce a dual-DT system designed for synergistic glioblastoma treatment. We first characterize the physical outputs of a single- versus dual-tube configuration, confirming that the simultaneous operation of two plasma tubes yields greater field strength and coverage without thermal damage to the surrounding media. We then evaluate the biological effects on human GBM cells (U87-MG), testing whether the dual-DT exposure produces supra-additive reductions in cell viability compared to one tube alone. Finally, we probe the underlying mechanisms of cell death via assays for intracellular ROS. U87-MG cells were chosen due to their established use as a standard glioblastoma model, with significant data present in the literature from being used with other plasma devices. Our central hypothesis is that the dual-plasma discharge tube system will induce more potent electromagnetic-induced oxidative stress in GBM cells than a single-tube system, leading to increased cell death through combined pathways. If validated, this dual-DT approach could lay the groundwork for a new non-invasive therapy for glioblastoma, offering a new treatment against this aggressive form of cancer.

## 2. Materials and Methods

### 2.1. Device Design and Configuration

The core plasma generation apparatus used in this study is a cold atmospheric plasma discharge tube (DT), which consists of a sealed dielectric vessel filled with helium gas. The plasma discharge tube (DT) devices are sealed quartz tubes initially purged with helium to displace ambient air. Once sealed, no gas exchange occurs during treatment. Each DT device comprises a cylindrical fused quartz cavity measuring 43 mm in outer diameter and 60 mm in length, with a wall thickness of 0.5 mm. A central stainless-steel electrode serves as the high-voltage conductor and is secured using a thin layer of silicone lubricant on the external surface to prevent gas exchange. The outer electrode is formed by wrapping the exterior of the quartz tube in copper foil (3 mm wide band of copper tape at upper flange of DT). Helium gas is introduced at a rate of 4 L/min for two minutes prior to discharge initiation in order to displace ambient air and ensure the purity of the plasma-forming medium. The plasma discharge is initiated and sustained using a 12.5 kHz sinusoidal alternating current (AC) waveform, which is generated by a function generator and then amplified by a flyback transformer. This setup yields a peak-to-peak voltage of approximately 10 kV with a transformer input of 10 V and 2 A.

For experiments involving dual DTs, two identical devices were mounted in a custom-designed platform resembling a helmet. This configuration permitted angular adjustment of each tube, enabling precise control over the spatial orientation of their respective emissions. The DTs could be aligned either perpendicularly (90 degrees apart) or directly opposite one another (0 degrees apart), with variable radial distances (r) and vertical heights (z) from the culture surface. This configurability allowed us to explore how the geometry of device placement influenced field overlap and cytotoxicity.

**Figure 1:**
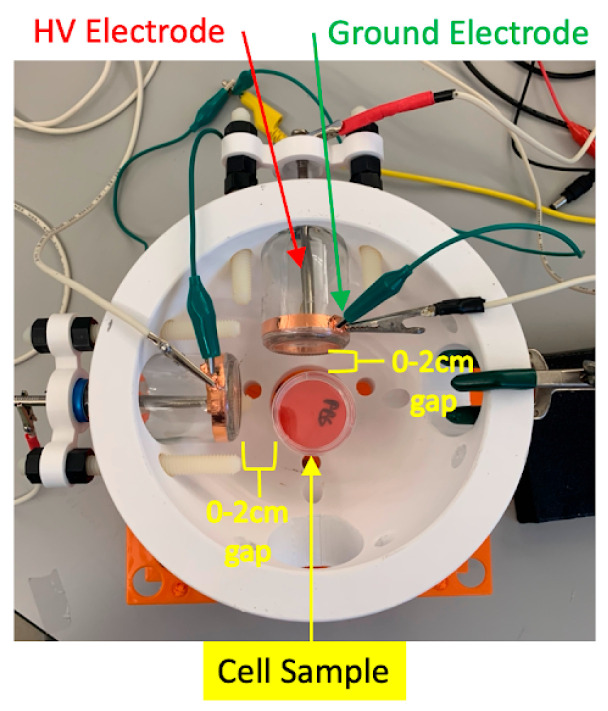
Diagram of the dual-plasma discharge tube helmet used for in vitro treatment of glioblastoma cell cultures. The platform allows for dynamic repositioning of both DT devices to optimize overlap of electromagnetic fields in the treatment zone.

**Figure 2:**
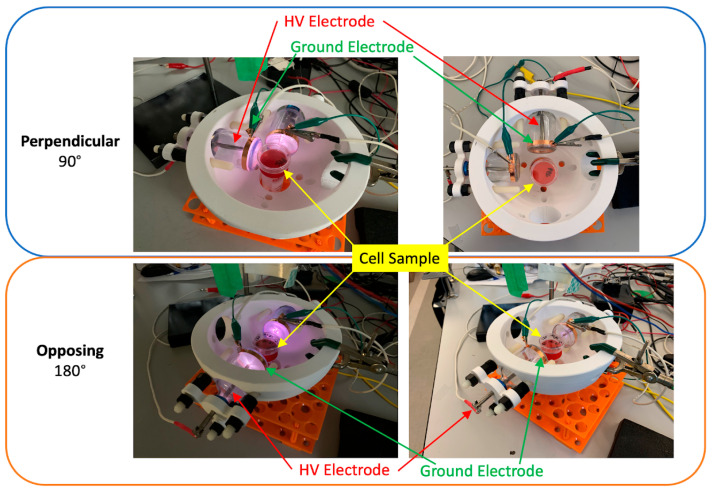
Comparison of 90 degree (**top**) and 0 degree (**bottom**) configurations of two DTs in the helmet mounting platform. Devices are shown ON (**left**) and OFF (**right**).

Figure 1 and Figure 2 show a schematic of the dual-DT helmet platform used for in vitro cell treatment. The schematic illustrates the relative positioning of the two discharge tubes and the culture dish positioned equidistant between them. The adjustable configuration enabled experimental control over spatial alignment, distance, and angular separation to test hypotheses about electromagnetic field interaction.

### 2.2. Electromagnetic Field Measurements

Time-varying electric and magnetic fields generated by the discharge tubes were quantified with two complementary instruments. Near-field waveforms were captured with a Tektronix TPP0850 passive voltage probe (Beaverton, OR, USA) (DC–800 MHz) and a Tektronix TCP0030A current probe (Beaverton, OR, USA) (DC–120 MHz) connected to a Tektronix MDO3014 mixed-domain oscilloscope (Beaverton, OR, USA) (1 GHz, 5 GS s^−1^). The probe tips were positioned at the geometric center of the treatment zone, 1.0 cm above the culture surface and equidistant from the two tubes during dual-DT operation. For each configuration, the oscilloscope recorded five successive 10 ms sweeps that were subsequently averaged. Absolute field strengths were obtained with a broadband MESTEK EMF01 electromagnetic-radiation tester (Shenzhen, China) (5 Hz–3.5 GHz; E-field resolution 0.1 V m^−1^; H-field resolution 0.01 µT) placed at the same location. The oscilloscope, probes, and EMF01 were factory-calibrated.

### 2.3. Cell Culture and Experimental Protocols

All experiments were conducted using U87-MG glioblastoma cells obtained from American Type Culture Collection (ATCC, U-87 MG, HTB-14™). Cells were cultured in standard conditions using Dulbecco’s Modified Eagle Medium (DMEM) supplemented with 10% fetal bovine serum and 1% penicillin–streptomycin. Cultures were maintained in a humidified atmosphere at 37 °C and 5% CO_2_. For all experiments, cells were seeded at a density of 5 × 10^5^ cells/mL into either 35 mm culture dishes or 96-well plates, depending on the requirements of the specific assay. Prior to plasma treatment, the media was replaced with 100 µL of fresh DMEM to ensure consistency in nutrient and pH conditions during exposure.

For plasma treatment, both single- and dual-DT configurations were used. The culture vessel was centered between the two discharge tubes at the same vertical distance. Treatments were administered for durations of 4, 8, or 12 min. In some instances, treatments were performed at 1, 5, 10, and 15 min intervals to fully characterize the test range. The difference in treatment time was guided by the cell culture dishes used and previous results using those parameters. All control groups were handled identically except that the DTs were not ignited. Immediately prior to DT treatment, overnight cell culture medium was aspirated and a minimal layer of fresh media was added to prevent desiccation during treatment. Following DT treatment, fresh medium was added for overnight culture.

### 2.4. Cell Viability and Spatial Mapping Assays

To quantify the effects of plasma exposure on cell viability, we utilized the WST-8 colorimetric assay (Dojindo Laboratories, Rockville, MD, USA). Following treatment, 10 µL of WST-8 reagent was added to each well, and the plates were incubated at 37 °C for two hours to allow the reaction to proceed. Absorbance was measured at 450 nm using a ThermoFisher Multiskan GO microplate reader (Waltham, MA, USA). Results were normalized to untreated controls to account for plate-to-plate variability.

In addition to global viability assessments, spatial resolution of cytotoxic effects was achieved using 96-well plates, with each well effectively representing a spatial point under the treatment field. The location-specific viability measurements were used to construct heat maps of cell death across the plate surface. These spatial maps allowed for identification of zones exhibiting the highest cytotoxicity, which were then compared to the expected overlap zones based on single-DT treatment. This spatial mapping approach was used to determine additive or supra-additive effects.

### 2.5. Flow Cytometry for Mechanistic Analysis

To investigate the mechanistic basis of cell death induced by single- and dual-DT configurations, we employed multiparametric flow cytometry. Following treatment, cells were harvested via trypsinization, washed with phosphate-buffered saline (PBS), and stained with a combination of fluorescent probes. SYTOX Blue was used to assess membrane integrity and overall viability, while Annexin V-FITC identified early apoptotic events. TMRM staining was used to evaluate mitochondrial membrane potential, and Invitrogen CellROX Deep Red (Carlsbad, CA, USA) was employed to detect intracellular reactive oxygen species. Samples were analyzed on a BD Celesta flow cytometer (Franklin Lakes, NJ, USA), and data were processed using FCS Express 7 software. Gating strategies excluded debris and doublets, and subpopulations of viable, apoptotic, necrotic, and ROS-high cells were quantified. These flow cytometry analyses enabled a comprehensive assessment of the cellular stress and death pathways activated by plasma treatment.

## 3. Results

### 3.1. Thermal Characterization Confirms Non-Cytotoxic Heating Profiles

**Figure 3:**
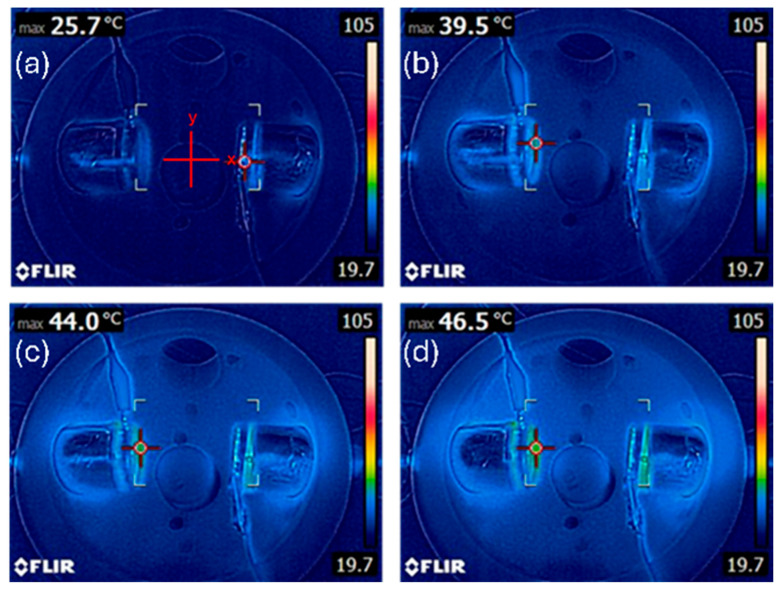
Thermal characterization of plasma discharge tube configurations. Temperatures recorded using infrared thermography confirm that both single- and dual-DT setups remain below cytotoxic thresholds at (**a**) 0 min, (**b**) 4 min, (**c**) 8 min, and (**d**) 12 min treatment times. Dual-DT setups show slightly elevated temperatures but still remain physiologically safe.

To determine whether any observed reduction in glioblastoma cell viability was attributable to thermal effects rather than plasma-induced electromagnetic stress, we first quantified the temperature profiles under each treatment condition. As can be observed in Figure 3, the infrared thermography and contact thermometer readings demonstrated that the temperatures remained below the cytotoxic threshold of 42 °C for all the treatment conditions. At the closest distance from the discharge tube (x = 1 cm), the temperature reached a maximum of 38.2 °C in the dual-DT setup following 12 min of exposure, compared to 37.5 °C in the single-DT condition. At x = 2 cm and beyond, the temperatures did not exceed 36 °C. These data confirm that the plasma-induced cytotoxicity in our experiments cannot be attributed to thermal insults.

### 3.2. Electromagnetic Field Mapping Reveals Constructive Interference in Dual-DT Configuration

Electromagnetic field measurements were performed to assess how the single- and dual-DT configurations differed in terms of field intensity and spectral complexity. Using voltage probes and an oscilloscope-based analysis, we quantified the electric (E-field) and magnetic (H-field) field components. In the dual-DT setup, the overlapping emissions generated areas of amplified field intensity directly between the two devices.

**Figure 4:**
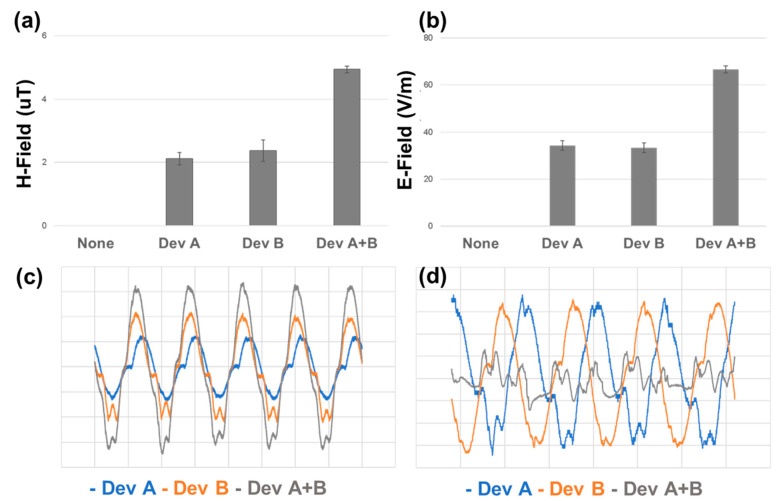
Electromagnetic field measurements for single- and dual-DT setups. (**a**) Magnetic (H-field) and (**b**) electric (E-field) field intensities are significantly enhanced in the dual-DT configuration. (**c**) Time-series near-field measurements show that in-phase operation of the dual-DT setup produces constructive interference, while (**d**) out-of-phase operation leads to destructive interference and attenuated field overlap. Pairwise comparisons of single DT compared to dual DT.

The synchronization of the two DTs was found to be a determinant of the field behavior. As can be seen in Figure 4, when driven in-phase, the dual setup produced constructive interference, resulting in significantly greater field intensities. Conversely, when the two DTs were operated with a 90-degree phase offset, destructive interference was observed, attenuating the central field strength. This is most obviousl in comparing the time-varying signal showing the constructive pattern in Figure 4c and the destructive pattern in Figure 4d.

### 3.3. WST-8 Assay Demonstrates Synergistic Cytotoxicity in Dual-DT Treatments

**Figure 5:**
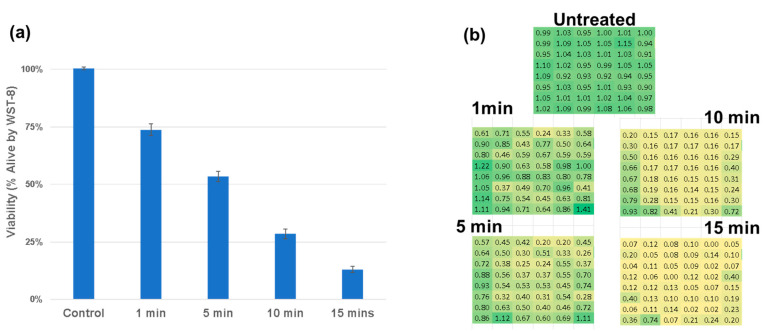
Dose-response analysis of cell viability following treatment with single- and dual-plasma discharge tubes. (**a**) WST-8 viability measurements show a time-dependent reduction in viability. (**b**) Spatially resolved heatmaps depict zones of cytotoxicity across the 96-well plate, with enhanced cell death observed in dual-DT overlap regions. A linear dose response is observed with R2 = 0.92.

To assess the biological effects of plasma exposure, we first conducted WST-8 viability assays on U87-MG glioblastoma cells. The treatment with a single-plasma discharge tube resulted in modest but statistically significant reductions in cell viability, as observed in Figure 5a. In contrast, the dual-DT configuration led to greater reductions, particularly in the regions corresponding to the predicted overlap of the electromagnetic fields. The spatial heatmaps in Figure 5b demonstrate the spatial diffusion of the treatment providing important dose-dependent context for treatment planning.

**Figure 6:**
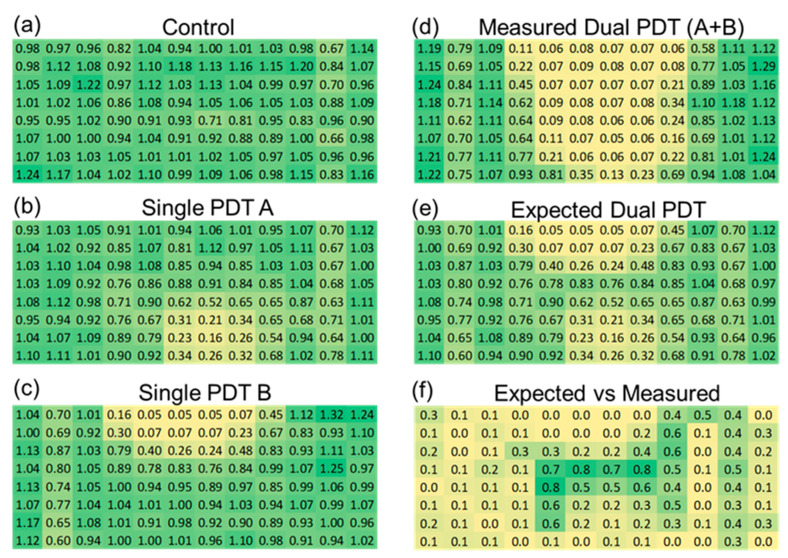
Spatial analysis of plasma-induced cytotoxicity. (**a**) Untreated control. (**b**) Single-DT A from +45 degrees. (**c**) Single-DT B from −45 degrees. (**d**) Dual-DT treatment. (**e**) Predicted additive model. (**f**) Differential heatmap showing statistically significant regions where dual-DT outcomes exceed additive predictions, demonstrating synergistic interactions in green.

As shown in the spatial heatmaps of viability in Figure 6a–c, the single-DT treatments reduced viability primarily in the wells directly beneath the emission axis, while the dual-DT treatment in Figure 6d created a broader, more diffuse zone of cytotoxicity. To evaluate the synergistic response of the dual device, we calculated the expected dual discharge response assuming additivity of the single device treatment from the data in Figure 6b,c. We then measured the expected additive dual discharge response (Figure 6e) to the true measured dual discharge responses (Figure 6d). As shown in Figure 6f, we demonstrate synergy of the two devices in the center of the treatment region, where the emissions of the two devices intersect.

**Figure 7:**
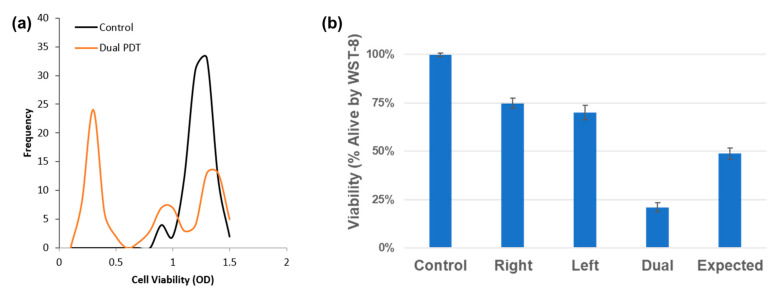
WST-8 viability assays confirm synergistic cell death in dual-DT treatments. (**a**) Population shift analysis shows a drop in viable cells. (**b**) Dual-DT treatment outcomes are significantly lower than the additive model derived from single-DT responses.

To validate the results obtained with the WST-8 assay in plates, we performed standard WST-8 viability assays under identical treatment conditions in dishes. The results mirrored those observed before, demonstrating that the dual-DT treatment reduced cell viability beyond additive predictions. As can be observed in Figure 7, comparison between the measured dual-DT outcomes and the theoretical sum of individual single-DT effects confirmed a significant deviation, further supporting the presence of a synergistic interaction.

### 3.4. Discharge Tube Mechanistically Induces Intracellular ROS Generation

**Figure 8:**
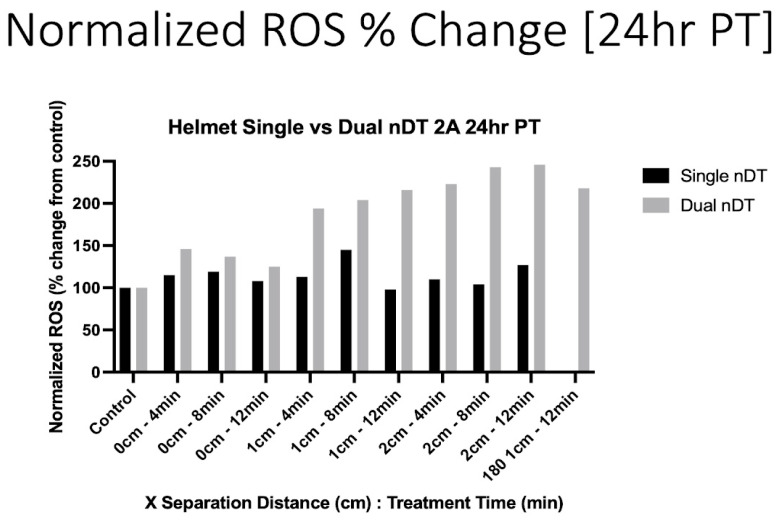
Intracellular ROS increases 24 h following single- or dual-DT treatment. Separation distances of 1 and 2 cm show larger changes when two DTs are in operation.

To assess the cellular effects leading to the observed cell death following the DT treatment, intracellular ROS was quantified by flow cytometry. Twenty-four hours after the DT treatment, the intracellular (IC) ROS concentrations were elevated after both the single- and dual-DT treatments. As can be seen in Figure 8, most of the single-DT conditions showed a mild increase in IC ROS, with a maximum of 140%, compared to the control, at 1 cm for an 8 min treatment. The dual-DT treatment caused a large increase in IC ROS for all treatment conditions, especially at 1 and 2 cm. The single opposing (0°) dual-DT configuration sample also displayed highly elevated IC ROS levels (200%), roughly the same as the perpendicular configuration for the 1 cm, 12 min, dual-DT treatment.

These results demonstrate the ability to increase the treatment efficacy by the simultaneous operation of multiple devices. While the goal of developing these devices and platforms is to reduce cancer cell viability, the observed increases in IC ROS are indicative of oxidative stress, which may lead to cell death via multiple cell death pathways. These initial results agree with previous studies showing DT induced an increase in IC ROS in U87-MG glioblastoma cells [16,17]. However, the dual-DT helmet treatment at 1 and 2 cm caused a substantially higher increase in IC ROS (200–250%) than a single DT in the helmet (max 150%) or a single DT treating the cell dish from the bottom (130%) [16].

### 3.5. Angular and Phase Dependence of Dual-DT Synergy

**Figure 9:**
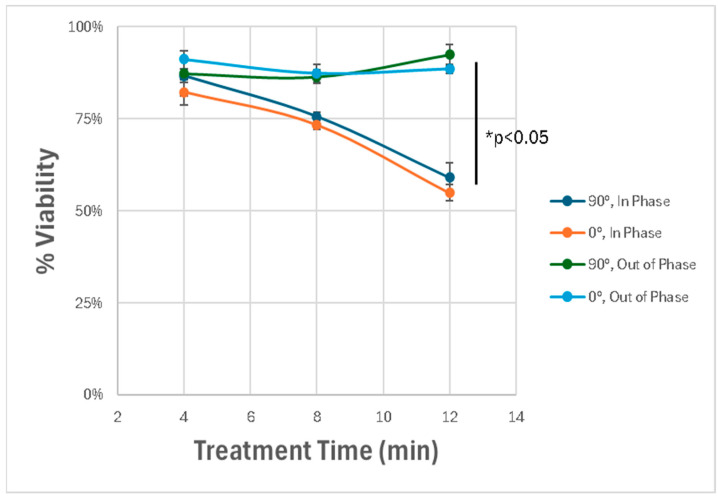
Effect of spatial orientation and phase alignment on cytotoxicity. Dual-DT setups exhibit maximal cytotoxicity only when operated in-phase. Out-of-phase devices fail to induce comparable effects, highlighting the role of constructive electromagnetic interference. * *p* < 0.05.

We next explored how the spatial orientation and phase synchronization of the discharge tubes influenced the therapeutic outcome. By varying the angle between the two DTs (0° vs. 90°) and adjusting their operating phase (in-phase vs. out-of-phase), we observed that only the in-phaseconfiguration induced significant cytotoxicity. When the tubes were out of phase to one another, the degree of cell death was substantially reduced, highlighting the importance of field interference patterns in generating synergistic effects. The angle of the device did not impact results. As can be seen in Figure 9, a dose dependent response can be observed and a statistically significant difference between in-phase and out-of-phase treatments.

These results demonstrate that the dual-DT platform induces enhanced glioblastoma cell death through synergistic, non-thermal mechanisms involving overlapping electromagnetic fields. The spatial precision and amplitude of these effects depend strongly on the device configuration, phase synchronization, and treatment duration.

## 4. Discussion

This study demonstrates that dual-plasma discharge tube (dual-DT) configurations amplify cytotoxic effects on U87-MG glioblastoma cells relative to single-DT setups. By systematically examining thermal, electromagnetic, and biological outcomes, we provide evidence that overlapping electromagnetic (EM) fields and heightened oxidative stress from complex EM interference underlie the observed synergistic cytotoxicity. These findings enable the development of advanced plasma-based therapies for glioblastoma [32].

The dual-DT treatment produced greater reductions in cell viability than the single-DT exposure, as shown by the WST-8 viability assays and flow cytometry. The spatially resolved WST-8 data indicated that the regions of maximal cell death coincided with the zones where the EM fields from the two tubes overlapped. The viability reduction achieved with the dual-DT device exceeded the predicted sum of two single-DT exposures, confirming a supra-additive interaction.

This synergy appears to arise from two primary factors: (1) the increased amplitude and broadened frequency spectrum of the EM fields in the dual-DT setup, and (2) the cumulative generation of intracellular reactive oxygen species (ROS). A Fourier analysis confirmed that the dual-DT operation yielded broader EM spectral peaks and higher field intensities than a single DT, which likely enhanced the cellular disruption. Consistently, the flow cytometry analysis showed intracellular ROS levels increasing by ~200% under the dual-DT treatment, compared to ~130% with the single-DT setup. This elevated ROS burden can overwhelm cellular antioxidant defenses, leading to oxidative damage, loss of mitochondrial function, and ultimately cell death [33].

Constructive interference between the two plasma sources is responsible for the intensified EM fields observed in the dual-DT configuration. The overlapping fields may induce perturbations in cellular structures, thereby compounding the cytotoxic effect. The plasma emissions span a broad spectrum of frequencies from kilohertz to terahertz, enabling multiple modes of interaction with cellular components. This contrasts with Tumor Treating Fields (TTF), a clinical therapy that uses narrow-band intermediate-frequency (~100 to 300 kHz) electric fields to disrupt mitotic spindle formation in dividing glioblastoma cells [17]. By affecting both dividing and non-dividing cells, the dual-DT system could therefore target the heterogeneous cell populations characteristic of glioblastomas more effectively.

While the single-DT exposure also induced cell death, its effects were confined to the cells directly beneath the discharge and dropped off rapidly with distance from the source, as would be expected with spatial EM field strength decay. In contrast, the dual-DT setup expanded the effective treatment area: the overlapping fields from the two tubes sustained higher EM amplitudes over a broader region. This extended reach addresses a key limitation of single-DT systems, which struggle to treat larger or diffuse tumor regions. In practical terms, dual-DT can deliver a more uniform cytotoxic effect across the target area.

The enhanced coverage and efficacy of the dual-DT approach support the feasibility of developing multi-tube plasma arrays for clinical applications. For example, a helmet-like device incorporating multiple DT units could be designed to treat intracranial tumors, providing the ability to shape and synchronize the EM fields in three dimensions around a glioma. Such a system would enable the precise targeting of tumor tissue while minimizing the exposure to the surrounding healthy brain tissues, and its non-invasive nature aligns well with the need for minimally invasive options in glioblastoma management. Ongoing research in our lab on the control systems for our plasma devices will allow the real-time tuning of the plasma chemistry, which is responsible for many of the EM emissions, particularly the high frequency emissions (GHz, THz) corresponding to the plasma frequency. Integrating feedback sensors could further improve the design of the setup into an intelligent plasma therapy platform that dynamically modulates the treatment parameters in response to real-time measurements for the optimization of efficacy and safety parameters.

Despite these promising results, several limitations must be addressed before translating dual-DT therapy toward clinical use. First, this study was performed entirely in vitro using a glioblastoma cell line. While U87-MG cells are a well-established model, they do not capture the full complexity of an in vivo tumor microenvironment. Currently, in vivo mouse PDX studies have only been performed with a single DT, but they demonstrate promising results in the enhanced survival of the implanted animals and in the reduction in the implanted tumor size when the DT was used as the only treatment or in combination with TMZ [21]. Future studies should evaluate dual-DT efficacy and safety in animal models, which will provide insight into issues such as the tissue attenuation of EM fields, ROS diffusion and scavenging in tissues, and any systemic or immunological effects of the treatment.

Secondly, the spatial and temporal parameters of the dual-DT system require further optimization. Variables such as the placement and orientation of the tubes, the phase alignment of their discharges, and the treatment duration may all influence outcomes. Systematically tuning these parameters could maximize tumor cell killing while minimizing off-target effects on normal cells. Implementing real-time monitoring (for instance, using OES or EM field mapping during treatment) in future prototypes could allow the dynamic adjustment of the plasma output to maintain effective dosing within safe limits. To expand on our empirical field measurements, finite-element simulations of the dual-DT geometry are currently underway. These three-dimensional models will resolve the spatial distribution of the electric and magnetic vectors within the treatment region and will be reported in a separate study.

Another practical limitation lies in the durability and consistency of the plasma discharge tubes themselves. Prolonged high-power operation can cause electrode erosion or the deposition of material onto the electrodes, which may alter the discharge characteristics over time in unpredictable ways. Future designs will investigate the use of other electrode materials or self-cleaning mechanisms to ensure stable performance and extend device lifespan, as well as components for single use whereby the electrode runtime would not be a consideration. Although we confirmed that the thermal effects were negligible in our in vitro setup (the median temperatures remained well below the cytotoxic thresholds), the potential for localized heating in vivo must be carefully examined. While this is challenging for the broad-spectrum EM emissions mentioned above, it is possible with advanced measurements of EM spectral components and power density that can be used to determine the specific absorption rate (SAR) of an individual EM frequency’s power density on a particular material or tissue type [34,35]. Performing specific absorption rate (SAR) measurements in tissue and thermal simulations will help to establish safe operating conditions, especially in the brain where heterogeneous tissue properties could lead to localized hotspots.

Our current findings identify elevated ROS and mitochondrial collapse as key mediators of dual-DT cytotoxicity, but the downstream molecular events warrant further exploration. Investigating markers of DNA damage (e.g., γH2AX for double-strand breaks), lipid peroxidation, and protein oxidation in dual-DT treated cells would deepen our understanding of how plasma-induced stress translates into cell death and may reveal additional therapeutic targets to exploit. Given the cellular heterogeneity of glioblastoma [36,37,38], future work should assess the differential responses of various tumor cell subpopulations to dual-DT treatment. High-content single-cell analyses could delineate whether certain resilient subpopulations survive the plasma exposure, guiding the development of combinatorial strategies to eliminate all tumor subsets. Previous research on the single-cell analysis of glioblastoma has elucidated unique mechanisms and pathologies for further treatment development [39,40].

Finally, while this study demonstrates the advantages of a dual-DT setup, the concept can be expanded to multi-DT arrays to tackle larger or multifocal tumors. A multi-tube configuration (e.g., an array of plasma emitters surrounding the tumor site) with programmable discharge parameters and feedback control could deliver treatment that is spatially tailored to each patient’s tumor geometry. By modulating factors such as the phase, frequency content, and gas composition of each plasma unit in real time, such a device could provide truly personalized plasma therapy for glioblastoma. This is similar to the methods developed for radiation therapy, where the intensity, shape, and other parameters of the treatment beam are modulated to spare healthy tissue and focus on the tumor area [41].

This information can be used in future studies to optimize the cancer killing capacity while minimizing the detrimental effects of DT treatment on normal healthy cells. With these advancements, dual-DT and next-generation multi-DT systems have the potential to overcome the limitations of current glioblastoma treatments and ultimately revolutionize the field of plasma oncology.

## 5. Conclusions

This study establishes that dual-plasma discharge tube configurations produce a significant, synergistic reduction in glioblastoma cell viability through mechanisms that are distinct from thermal cytotoxicity and instead driven by overlapping electromagnetic fields and elevated oxidative stress. By tuning the spatial emissions from two synchronously operated DTs, the treatment system creates a field environment that exceeds the physical and biological impact of individual tubes, enabling a more comprehensive disruption of cellular homeostasis. The enhanced efficacy is linked to constructive electromagnetic interference and increased intracellular ROS generation, both of which contribute to mitochondrial dysfunction and the activation of cell death pathways.

Our findings validate the concept of using multiple spatially coordinated plasma sources for therapeutic applications while also highlighting the importance of precise control over device placement, orientation, and phase synchronization. The helmet-like architecture introduced here offers a scalable framework for future multi-DT systems that could be adapted to the anatomical complexity of glioblastoma and other solid tumors.

These results offer an alternative to conventional electric-field-based modalities such as Tumor Treating Fields. Unlike TTF, which primarily disrupt mitosis, dual-DT treatment exerts cellular stressors capable of targeting both dividing and non-dividing tumor populations, including those in hypoxic or dormant states. This helps establish a new paradigm of multi-DT plasma therapy as a novel approach within the field of plasma oncology.

While further validation using in vivo models is required, this work establishes the preliminary designs for the continued development of intelligent, array-based plasma systems that are spatially adaptable, mechanistically multifactorial, and non-invasive. With the appropriate optimization and integration into clinical workflows, such devices may represent a novel paradigm for treating glioblastoma.

## Data Availability

Full datasets are available on request to the corresponding author.
